# The role of K^+^ channels in uptake and redistribution of potassium in the model plant *Arabidopsis thaliana*

**DOI:** 10.3389/fpls.2013.00224

**Published:** 2013-06-27

**Authors:** Tripti Sharma, Ingo Dreyer, Janin Riedelsberger

**Affiliations:** ^1^Molecular Biology, Institute for Biochemistry and Biology, University of PotsdamPotsdam, Germany; ^2^IMPRS-PMPG, Max-Planck Institute of Molecular Plant PhysiologyPotsdam, Germany; ^3^Centro de Biotecnologia y Genomica de Plantas, Universidad Politécnica de MadridMadrid, Spain

**Keywords:** plant potassium channel, *Shaker*, TPK, K_ir_-like, *Arabidopsis thaliana*, voltage-dependent, voltage-independent

## Abstract

Potassium (K^+^) is inevitable for plant growth and development. It plays a crucial role in the regulation of enzyme activities, in adjusting the electrical membrane potential and the cellular turgor, in regulating cellular homeostasis and in the stabilization of protein synthesis. Uptake of K^+^ from the soil and its transport to growing organs is essential for a healthy plant development. Uptake and allocation of K^+^ are performed by K^+^ channels and transporters belonging to different protein families. In this review we summarize the knowledge on the versatile physiological roles of plant K^+^ channels and their behavior under stress conditions in the model plant *Arabidopsis thaliana*.

## Introduction

Potassium (K^+^) is essential for growth and development of an organism. It is involved in various important cellular processes, like stabilization of protein synthesis, activation of enzymes, neutralization of negative charges on proteins and many more. In addition to the above mentioned tasks, in plants it is a key player in osmotic processes contributing to cellular turgor, cell elongation, translocation of photosynthates, maintenance of cytosolic pH homeostasis, and the setting of the membrane potential along with the proton motive force (Maathuis, 2009; Marschner, 2012). All these functions justify it being the most abundant inorganic cation in plants, contributing to up to 10% of their dry mass (Leigh and Wyn Jones, 1984).

Potassium is a major factor in resistance to drought, salinity, and fungal diseases (Amtmann et al., 2008). This explains why it is of crucial importance in agriculture affecting crop yield. For performing the tasks explained above, plants require potassium concentrations ranging between 100–200 mM in the cytoplasm (Wyn Jones and Pollard, 1983). In contrast, concentration of potassium in soil (10–100 μM) is 3–4 orders of magnitude lower (Schroeder et al., 1994). Therefore, a plant has to invest energy for the uptake of K^+^ and its distribution throughout the plant.

The transport of potassium is accomplished by a variety of transporter proteins. In the plant model organism *Arabidopsis thaliana* a total of 71 K^+^ channels and transporters have already been identified (Mäser et al., 2001; Véry and Sentenac, 2003; Amtmann et al., 2004; Wang and Wu, 2010). They have been categorized into six different gene families, comprising of three channel families and three transporter families (KUP/HAK/KT, HKT, and CPA families; Gierth and Mäser, 2007; Chanroj et al., 2012; Gomez-Porras et al., 2012).

**Figure 1:**
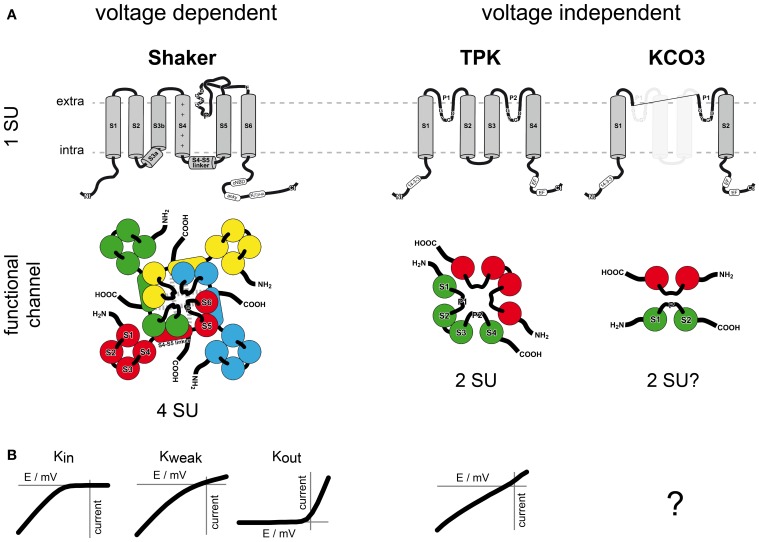
**Structure and function of K^+^ channel families in plants**. The two plant K^+^ channel families vary in **(A)** structure and **(B)** function. *Shaker* channels form the most versatile family among plant K^+^ channels. Nine members segregate into inwardly, outwardly and weakly rectifying channels. Functional channels are tetramers and operate in a voltage dependent manner. One subunit consists of six transmembrane domains (S1–S6) and one pore domain (P). The fourth transmembrane region S4 is rich in positively charged amino acids and acts together with S1, S2, and S3 as voltage sensor. Five TPK channels have been identified. One subunit contains two pore domains (P1 and P2) and two subunits are sufficient to form a functional channel. TPKs act in a largely voltage independent manner and exhibit leak like currents. KCO3 was initially classified as a K_ir_-like channel showing two transmembrane regions and one pore domain. In fact, “plant K_ir_-like channels” originate from TPKs by partial deletion of one selectivity-filter and two transmembrane domains. In line with this notion, only stable dimers have been detected. A K^+^ transport function has not been shown for these truncated channels. Abbreviations: extra, extracellular side; intra, intracellular side; SU, subunit; +, positively charged amino acids; cNBD, cyclic nucleotide binding domain; anky, ankyrin repeat domain; K_(T)/HA_, acidic domain; EF, EF hand domain.

The three identified families of K^+^ channels are *Shaker*, Tandem-Pore K^+^ (TPK) and K_ir_-like channels. Recent phylogenetic data, however, evidenced that K_ir_-like channels in fact belong to the TPK family and originated by evolutionarily recent gene duplication and partial deletion events (Marcel et al., 2010; Voelker et al., 2010; Gomez-Porras et al., 2012). We therefore do not consider K_ir_-like channels as a separate family anymore. K^+^ channels are active as multimeric proteins composed of two or four α-subunits, which are characterized by the presence of either one or two pore (P) domains. In the functional multimeric protein, four P domains are associated to form part of the conduction pathway, including its selectivity filter. K^+^ selective channels have the hallmark motif TXGYGD/E in their P domains (Lebaudy et al., 2007; Table 1, Figure 1).

**Table 1:**
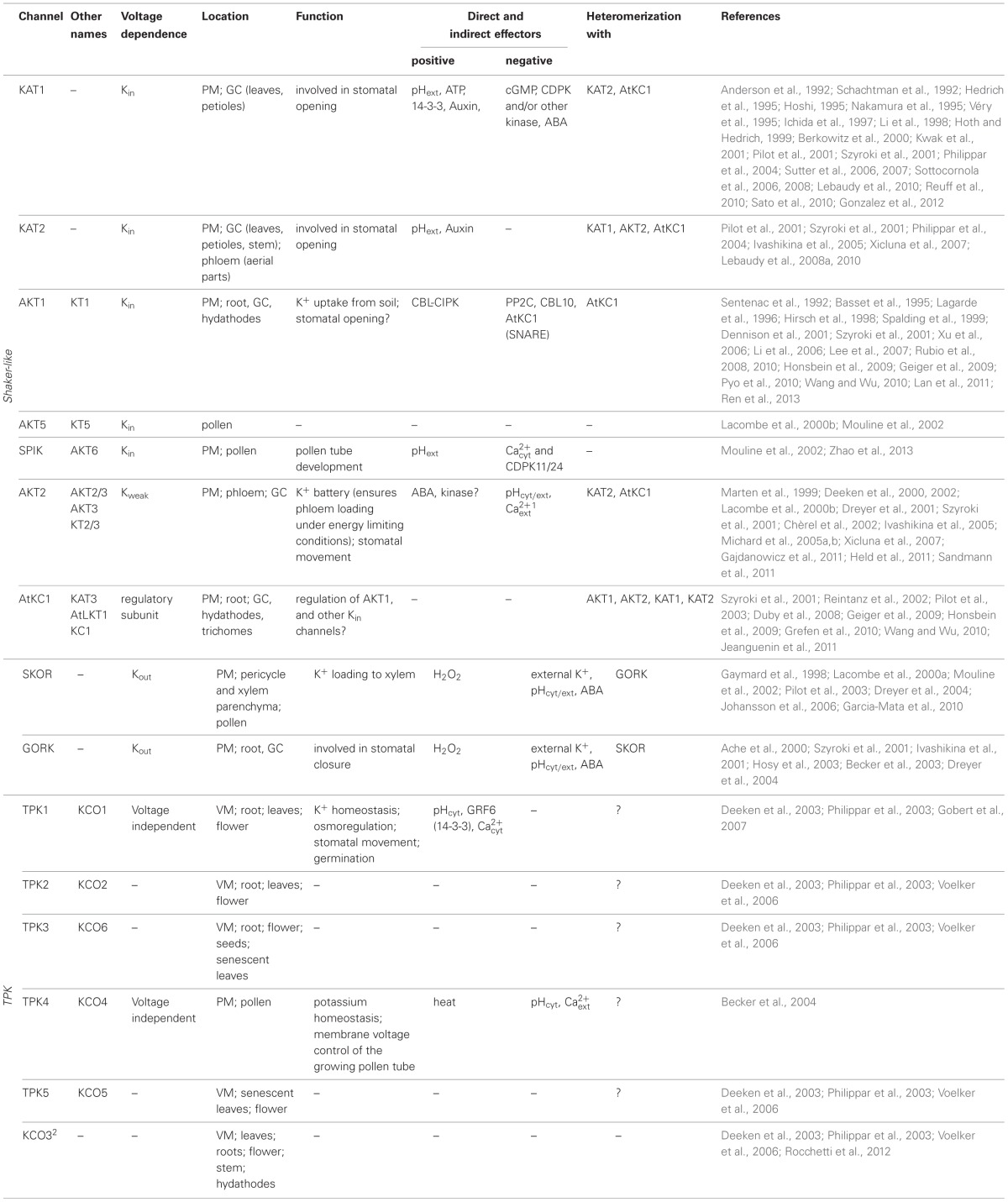
**Overview of location, function and regulation of plant K^+^ channels**.

All pH data refer to pH acidification. Abbreviations: K_in_, K_weak_, K_out_, inwardly, weakly, outwardly rectifying potassium channel; PM, plasma membrane; VM, vacuolar membrane; GC, guard cells; CDPK, Ca^2+^ dependent protein kinase; ABA, abscisic acid; CBL, calcineurin B-like calcium sensors; CIPK, CBL-interacting protein kinase; PP2C, 2C-type protein phosphatase; cyt, cytosolic; ext, extracellular.

^1^only inward currents are affected.

^2^KCO3 very likely evolved from TPK2 by gene duplication and a subsequent deletion of one of the two pore regions.

In 1992, AKT1 and KAT1, two inward rectifying channels from *Arabidopsis* were identified by functional complementation of yeast strains defective in potassium uptake. These two members of the *Shaker*-like channel family were the first cloned plant potassium channels (Anderson et al., 1992; Schachtman et al., 1992; Sentenac et al., 1992).

In 1997, a first member of the TPK channel family was identified by *in silico* approaches, utilizing the *Arabidopsis* gene sequencing program. TPK channels are the plant counterparts of animal Tandem Pore (TWIK-like) channels (Czempinski et al., 1997). While searching for TPK1-related sequences in genome sequence database, KCO3 was identified and was thought to be structurally similar to animal potassium inward rectifying channels (Czempinski et al., 1999) leading to its initial classification into a separate family of plant K_ir_-like channels.

## Voltage independent K^+^ channels in *Arabidopsis*

### Tandem Pore potassium channels

The Tandem-Pore K^+^ (TPK) channel family comprises six members (TPK1-TPK5 and KCO3, see also below for this special case) in the model plant *Arabidopsis thaliana*. TPK homologues were identified in higher plants and green algae (Voelker et al., 2010; Gomez-Porras et al., 2012). A phylogenetic analysis has shown that plant TPK channels are divided into two subfamilies: TPK1 belongs to one and TPK2, TPK3, TPK4, and TPK5 to the second subfamily. This sub division in families indicates a common ancestral origin of the channels TPK2, TPK3, TPK4, and TPK5; a hypothesis that was further supported by the analysis of chromosome segment duplication in the *Arabidopsis* genome (Marcel et al., 2010; Voelker et al., 2010; Gomez-Porras et al., 2012).

The first TPK channel (AtTPK1) was cloned via an *A. thaliana* EST database search for the conserved K^+^ channel pore domain motif TXGYGD (Czempinski et al., 1997). TPKs show a TM-P-TM-TM-P-TM structure with a duplicated transmembrane-pore-transmembrane module (Figure 1A). In general, these channels contain one or two Ca^2+^-binding EF hands in the cytosolic C-terminal part and binding sites for 14-3-3 proteins in the cytosolic N-terminal part, as well as a putative N-glycosylation site in the luminal loop between the pore domain and the second transmembrane domain.

Functional TPK channels are built of two of such subunits and exist as dimers (Maitrejean et al., 2011). They show a high Ca^2+^ dependency, which might be important for channel regulation (Latz et al., 2007a). TPK channels have been localized in the vacuolar membrane (Czempinski et al., 2002; Schönknecht et al., 2002). One exception is TPK4, which has been reported to localize in the plasma membrane (Becker et al., 2004; Dunkel et al., 2008). TPK4 shares 85% similarity (53% identity) with TPK5 but lacks the regulatory domains and the 14-3-3 protein interaction motif. It might thus be speculated that TPK4 evolved from TPK5 and subsequently underwent truncation events. Another exception from exclusive vacuolar localization might be TPK3. In Western blots TPK3 was also identified in thylakoid membranes (Zanetti et al., 2010) raising the question whether TPKs may have multiple subcellular locations.

Expression analysis of TPKs through quantitative real-time PCR experiments evidenced their presence in different plant tissues like roots, leaves and flowers (Deeken et al., 2003; Voelker et al., 2010). Among all TPKs, *TPK1* showed the highest expression levels in all tissues analysed, followed by *TPK3* and *TPK5*. Expression levels of *TPK2* and *TPK4* were very low. Elevated levels of *TPK2* transcripts were detected in stamen and pollen. *TPK3* transcript levels were more abundant in petals, stamen, seeds and senescent leaves.

#### Assembly status of Tandem-Pore K^+^ channels

Promoter-reporter gene studies and qRT-PCR experiments revealed overlapping expression patterns for members of the TPK/KCO3 channel family (Czempinski et al., 2002; Deeken et al., 2003; Philippar et al., 2003; Becker et al., 2004; Voelker et al., 2006). Expression of *TPK1* overlaps with that of *TPK3* in root tips and with that of *TPK5* and *KCO3* in vascular tissues. Additionally, *TPK1, TPK2, TPK3*, and *TPK4* express in pollen. The overlapping expression patterns and their common localization in the tonoplast propose that heteromeric channel subunit combination might occur under different developmental stages or physiological conditions (Latz et al., 2007a).

Dimerization of TPK channels has been shown experimentally by using velocity sucrose gradient centrifugation of leaf homogenates expressing TPK1-GFP. This confirms the contribution of four pore domains to the K^+^ selectivity filter of the TPK1 channel (Maitrejean et al., 2011). Using the same technique, AtKCO3 and AtKCO3-GFP have been observed to exist as dimers, too. These channels would thus have only two pore domains in a dimerized state (Figure 1A), which is not considered to be sufficient for an active, K^+^-selective channel (Rocchetti et al., 2012).

With the aim of studying the assembly status of TPK/KCO family members, various experiments have been performed employing techniques like FRET and BiFC (split-YFP). Results from these approaches indicated the existence of homomeric TPK/KCO3 channels, as e.g., in the case of TPK1 or TPK5 (Voelker et al., 2006). However, so far no evidence for heteromeric channel formation could be provided. Nevertheless, there are neither convincing data ruling out this possibility. Thus it cannot be excluded that *in vivo* heteromeric channel formation might occur under different developmental and physiological conditions.

#### Localization of Tandem-Pore K^+^ channels

In a first approach to detect the subcellular localization, the TPK1 channel has been stably over-expressed in tobacco BY-2 cells. After protein fractionation with a sucrose gradient, this K^+^ channel was found to co-fractionate with tonoplast markers, giving a first clue of its localization on the vacuolar membrane (Czempinski et al., 2002). Further localization studies were performed by creating GFP fusion constructs followed by their transient expression in *A. thaliana* protoplasts. Such experiments demonstrated vacuolar localization of TPK1, TPK2, TPK3, and TPK5 (Voelker et al., 2006). In contrast, when a TPK4:GFP fusion construct was expressed in onion epidermal cells, it was found to localize partially in the plasma membrane. A major fraction, however, was detected in the ER (Becker et al., 2004; Dunkel et al., 2008). This might be either due to ER-retention or may indicate that besides TPK3, been found in the tonoplast and in the thylakoid membrane, also TPK4 may exhibit at least a dual localization profile.

Unfortunately till now, no general targeting sequence is known that “guides” TPK channels to the appropriate membrane (Vitale and Hinz, 2005; Dunkel et al., 2008). With the purpose of identifying the sorting signal of vacuolar TPK channels, various chimeras were generated between TPK4 (plasma membrane protein) and TPK1 (tonoplast protein). It is not handed down why this particular pair has been chosen and not the “twins” TPK5 and TPK4; TPK4 sharing 85% of similar amino acids with TPK5. Nevertheless, the chimeras showed that complete replacement of the cytosolic C-terminus of TPK1 results in ER retention. Further detailed analysis indicated that the terminal 25 amino acids are not important for the trafficking process. An analysis of amino acids 292–308 in the C-terminus of TPK1 could identify three diacidic motifs. Out of these three motifs, mutations in (D296G/E298G) resulted in ER-stuck TPK1 proteins, suggesting that this diacidic motif is crucial for the export of TPK1 from the ER (Dunkel et al., 2008; Voelker et al., 2010). A related study on rice TPKs identified amino acids in the cytosolic C-terminal domain that determine differential targeting of TPKs to the endomembranes of the large central lytic vacuole or of protein storage vacuoles (Isayenkov et al., 2011a) indicating a general role of certain regions in the cytosolic C-terminus for channel targeting.

Retention of TPK1 channel protein in the ER also occurred when plant leaves were treated with Brefeldin A, a fungal toxin which causes redistribution of Golgi membranes. From this observation it was inferred that the transport of TPK channel proteins to the vacuolar membrane is through a Golgi-dependent pathway and that the Golgi apparatus is the first compartment crossed by the protein after it leaves the ER (Dunkel et al., 2008). Experiments in rice indicated a more complex situation of TPK targeting. TPKs targeted to the lytic vacuole indeed cross the Golgi apparatus. However, TPKs targeted to protein storage vacuoles apparently reach the endomembrane in a Golgi-independent way (Isayenkov et al., 2011a).

#### Regulation and function of Tandem-Pore K^+^ channels

At present the knowledge on function and regulation of plant TPKs is limited. Research is often fuelled by comparison with related channels from other kingdoms. Animal two-pore channel activity has been shown to be regulated by interacting 14-3-3 proteins (Rajan et al., 2002). Also in plants down-regulation of K^+^ channel activity in the tonoplast has been observed to be caused by interaction with 14-3-3 proteins. In TPKs, the cytosolic N-terminus comprises a classical binding motif for 14-3-3 proteins (RSXpS/pTXP)^1^. Phosphorylation of these serine or threonine residues is crucial for the interaction with 14-3-3 proteins (Latz et al., 2007a).

TPK channels are proposed to be involved in the K^+^ homeostasis of plant cells by allowing the controlled intracellular K^+^ transport from and into organelles. Recent experiments employing the patch clamp technique have demonstrated a mechano-sensitive nature of TPK channels suggesting especially a role in osmoregulation. This concept was further supported by protoplast disruption assays (Maathuis, 2011) and seedling germination tests (Gobert et al., 2007).

AtTPK1 is ubiquitously expressed in *A. thaliana*. Using promoter-reporter gene (GUS) fusion, *TPK1* promoter activity was observed in root cortex, vascular tissue, mesophyll cells, guard cells and pollen grains (Czempinski et al., 2002). When expressed in yeast, TPK1 has characteristics of K^+^-selective channels from *Vicia faba* (VK channels) previously characterized *in vivo* with strong selectivity for K^+^ over Na^+^ (Bihler et al., 2005; Gobert et al., 2007; Latz et al., 2007b). The activity of TPK1 is independent of the membrane voltage but was shown to be dependent on the cytosolic pH with a maximum open probability at pH 6.7, decreasing 20–30% at physiological pH 7.5–7.8. It is activated by cytosolic Ca^2+^, remarkably exhibiting the highest affinity for calcium ions among the proteins tested including calmodulin. Interaction of TPK1 with the 14-3-3 protein GRF6 (General Release Factor 6) increases the channel activity in a dose dependent manner. This interaction does not play any role in targeting of the protein to the tonoplast (Latz et al., 2007a). All these data indicate that TPK1 is tightly controlled by cellular signals. TPK1 has been reported to participate in vacuolar K^+^ release during stomatal closure and also during seed germination and radicle growth (Gobert et al., 2007).

AtTPK4 is an instantaneously activating, K^+^ selective channel that is also found in the plasma membrane when expressed in *Xenopus* oocytes and yeast. *In planta, TPK4* exhibits low transcript abundance. It is predominantly expressed in pollen, as observed by promoter-GUS fusion analysis. TPK4 is blocked by extracellular Ca^2+^ and is insensitive toward changes in extracellular pH, but it is efficiently blocked by cytosolic acidification. Activation of TPK4 by heat has also been reported (Becker et al., 2004). TPK4 is proposed to contribute to the K^+^ conductance of the pollen tube plasma membrane, where it operates as a so called “open rectifier” with saturating current at depolarizing membrane potentials.

AtTPK5 is targeted to the tonoplast. At the mRNA level, *TPK5* shows higher abundance in senescent leaves and petals (Voelker et al., 2010). Promoter GUS studies of *TPK5* have shown expression in the vascular tissues of leaves, roots, hydathodes, floral tissues and stems. *TPK5* transcript level is increased or decreased in response to external factors.

Recently AtTPK1, AtTPK2, and AtTPK5 were functionally characterized in *Escherichia coli*. The three isoforms were able to complement the K^+^ uptake deficient *E. coli* mutant LB2003 on low K^+^ medium (Isayenkov and Maathuis, 2013). Interestingly, in the same experiments AtTPK3 could not complement LB2003. This may indicate that this channel might be active in a different membrane environment, as for instance the thylakoid membrane (Zanetti et al., 2010).

#### Different isoforms of Tandem-Pore K^+^ channels

Tandem-pore K^+^ channels have also been identified and characterized in plant species other than *A. thaliana*, for example *Hordeum vulgare, Nicotiana tabacum, Solanum tuberosum, Oryza sativa* (Czempinski et al., 1999; Hamamoto et al., 2008a,b; Isayenkov et al., 2011a,b). It is fascinating to see that NtTPK1 from tobacco exhibits properties different from other plant TPK channels, since it is active even in the absence of Ca^2+^. Nevertheless, increase in cytosolic Ca^2+^ resulted in an up to two fold increase in the K^+^ current amplitude (Hamamoto et al., 2008a). Its current profile shows an instantaneous and a time-dependent component (Hamamoto et al., 2008b). The most interesting distinguishing feature is that two of the four identified isoforms in *N. tabacum* do not contain the conserved TXGYGD motif in the second pore domain. Instead, NtTPKb and NtTPKc possess VHG or GHG, respectively.

### Plant K_ir_-like channels

Plant K_ir_-like channels were initially classified as an own group although they are similar to TPK channels. To date, they have been found only in the genus *Arabidopsis, (A. thaliana* and *A. lyrata*; Gomez-Porras et al., 2012). Thus, they apparently emerged just recently in evolution. Phylogenetic analyses indicated them to have originated from gene duplication of an TPK channel gene followed by a partial deletion event that resulted in the loss of one pore domain (Figure 1A; Marcel et al., 2010; Voelker et al., 2010). As a consequence, a plant K_ir_-like channel subunit contains only two TM and one P region. Based on that structural feature it was speculated that plant K_ir_-like channels are tetramers. This concept, however, is rather questionable. The genome of *A. thaliana* contains only one gene (called KCO3) coding for a K_ir_-like subunit. Recently, KCO3 could be detected only as stable dimer at the biochemical level (Rocchetti et al., 2012) pointing further to its origin from TPK channels. Very low transcript abundance has been observed for *KCO3*. Promoter-GUS fusion constructs for *KCO3* show expression in vascular tissue of leaves, roots, flower tissue and stem and also in hydathodes as seen also for *TPK5*. KCO3 might play a role in osmoregulation, as the knock-out plant for the *KCO3* gene shows reduced growth under osmotic stress condition. However, this change in the plant phenotype can be complemented by expressing a mutant *KCO3* gene with an inactive pore region. These results indicate that the function of KCO3 under osmotic stress conditions is independent of its ability to transport potassium ions (Rocchetti et al., 2012). In conclusion, based on the current knowledge, plant K_ir_-like channels should be re-integrated into the TPK family, instead of being considered as a separate channel family. Their occurrence in *Arabidopsis*, only, may suggest that they just are “a freak of nature” without fundamental physiological importance outside this genus.

## Voltage dependent K^+^ channels in *Arabidopsis*

The so-called plant *Shaker*-family is a group of voltage gated K^+^ channels. In *A. thaliana* it comprises nine members. This group can be divided into three subfamilies regarding their response to the membrane voltage (Lebaudy et al., 2007; Dreyer and Blatt, 2009). Six members activate upon membrane hyperpolarization and are closed when the driving force for potassium is outwardly directed. As a consequence they elicit only inward K^+^ currents (K_in_). Two members activate upon membrane depolarization. They are closed when the driving force for potassium is inwardly directed. Thus, they elicit only outward K^+^ currents (K_out_). And one member exhibits weak voltage dependence and can mediate both, K^+^ efflux and K^+^ influx (K_weak_; Figure 1B).

Functional plant *Shaker* channels are built of four α-subunits. Each α-subunit contains six transmembrane domains and one pore domain between the fifth and the sixth transmembrane domain. The C-terminus contains various regulatory elements, like the cyclic nucleotide binding domain, an ankyrin repeat domain, the acidic domain K_HA_ and in K_in_ channels the K_T_ domain (Sentenac et al., 1992; Ehrhardt et al., 1997; Gaymard et al., 1998; Dreyer et al., 2004). Besides being functional as homotetramers, the formation of heterotetramers is common and proven to occur in plants (Dreyer et al., 1997; Lebaudy et al., 2008a).

Versatile physiological roles of plant *Shaker* channels were identified in numerous experiments. Knock-out and overexpressing mutant plants, as well as heterologous expression systems like *Saccharomyces cerevisiae, Xenopus laevis* oocytes, HEK293, COS, or Sf9 cells were used to study the functionality of K^+^ channels (Dreyer et al., 1999). The physiological roles and impacts of plant *Shaker* channels on the plant are described in the following sections.

### K^+^ uptake into roots via AKT1

#### Various conditions necessitate different uptake systems

**Figure 2:**
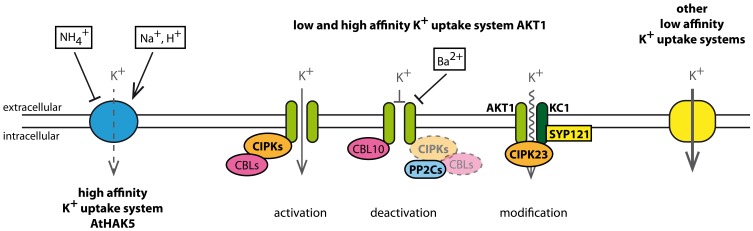
**K^+^ uptake into *Arabidopsis* roots and its regulation**. Depending on the actual K^+^ concentration in the soil different low or high affinity K^+^ uptake systems are active. At K^+^ concentrations below 0.01 mM only the high affinity transporter AtHAK5 is active. It is blocked by extracellular NH^+^_4_ and stimulated by extracellular Na^+^ and H^+^. The *Shaker*-like K^+^ channel AKT1 is involved in high and low affinity K^+^ uptake. It is a target of an extensive regulatory network that includes calcium sensors (CBLs), kinases (CIPKs), phosphatases (PP2Cs), and the ability to form heterotetramers with AtKC1. In the presence of CBL1 or CBL9, CIPK23 phosphorylates and activates AKT1. The interaction of CIPK 6, 16, and 23 each with CBL1, 2, 3, and 9 and its effect on AKT1 were shown in yeast two-hybrid assays and *Xenopus laevis* oocytes (Lee et al., 2007). AKT1 is deactivated by a direct interaction with CBL10, external Ba^2+^, or dephosphorylation via PP2C phosphatases. Phosphatases act directly on AKT1 or on the CIPK-CBL machinery to inactivate AKT1 (Lan et al., 2011). Furthermore, AKT1 is able to form heterotetramers with AtKC1. The heteromeric channel exhibits changed gating and permeation properties that block efficiently potential K^+^ release under low external K^+^ concentrations (Geiger et al., 2009). In addition, an interaction of CIPK23 with the heteromeric AKT1-AtKC1 was suggested and the contribution of SYP121 to the native characteristics of AKT1-AtKC1 was described (Honsbein et al., 2009).

K^+^ uptake from soil is performed by a well-organized system of transport proteins each contributing in its own manner (Alemán et al., 2011). All uptake systems together operate on a broad range of K^+^ concentrations and are part of an extensive regulatory network (Figure 2). As main K^+^ uptake systems in *Arabidopsis* roots the *Shaker*-like K^+^ channel AKT1 and the K^+^ transporter AtHAK5 have been identified (Hirsch et al., 1998; Gierth et al., 2005; Rubio et al., 2008). At external K^+^ concentrations below 0.01 mM the proton-driven H^+^/K^+^ co-transporter AtHAK5 is the only system responsible for K^+^ uptake from the soil. At K^+^ concentrations between 0.01 mM and 0.05 mM AtHAK5 and AKT1 together contribute to K^+^ uptake. At higher external K^+^ concentrations, AKT1 together with other unknown low affinity K^+^ uptake systems are responsible for K^+^ uptake from the soil (Rubio et al., 2010; Pyo et al., 2010; Caballero et al., 2012).

AKT1 and AtHAK5 are affected by different environmental conditions. Both transport proteins work at different K^+^ concentration spectra and exhibit individual sensitivity toward other ions. For instance, AtHAK5 is sensitive to ammonium (NH^+^_4_) whereas AKT1 remains unaffected in the presence of NH^+^_4_. On the contrary, Ba^2+^ blocks AKT1 while AtHAK5 remains unaffected, and Na^+^ and H^+^ stimulate activity of AtHAK5 (Hirsch et al., 1998; Spalding et al., 1999; Rubio et al., 2008). Therefore, the different K^+^ uptake systems complement one another and even permit K^+^ uptake when one uptake system is disabled. AKT1 provides hence an alternative K^+^ uptake system to the NH^+^_4_ sensitive AtHAK5 under low K^+^ conditions.

#### Regulation of AKT1

AKT1 itself contributes to high and low affinity K^+^ uptake and is target of a regulatory network. Xu et al. and Li et al. showed in 2006 that CIPK23 and CBL1 or CBL9 are required to activate AKT1. The two calcineurin B-like calcium sensors CBL1 and CBL9 bind to the CBL-interacting protein kinase CIPK23, which then in turn phosphorylates AKT1. All three components (AKT1-CIPK23-CBL1/9) are essential for a functional expression of AKT1 in oocytes of *X. laevis*.

Shortly after, further components of this highly complex and flexible regulatory network were discovered. Besides several CIP kinases a 2C-type protein phosphatase (PP2C), AIP1, was shown to bind and inactivate AKT1 (Lee et al., 2007). Subsequent studies detected more interrelations between several CBLs and CIPKs with AKT1 (Lee et al., 2007; Lan et al., 2011; Ren et al., 2013). Lan et al. suggested that PP2C phosphatases also interact with the CIPK-CBL complex to inhibit the phosphorylation activity of the kinase and to dephosphorylate AKT1. And Ren et al. (2013) demonstrated that CBL10 directly binds AKT1 and diminishes its activity in a concentration-dependent and CIPK-independent manner.

Many different associations of AKT1 with CBLs, CIPKs, and PP2Cs have been reported. Grefen and Blatt (2012) argue that the method used to investigate interactions between proteins, positioning of tags and the way of analysis have a decisive impact on detectable interactions. Nevertheless, CBLs, CIPKs and PP2Cs provide a comprehensive system to regulate the K^+^ uptake mediated by AKT1. Especially, as different CBLs are involved in different signaling pathways this phosphorylation-dephosphorylation system provides a powerful regulatory network for the plant to respond to a broad range of environmental changes (for review see Kudla et al., 2010).

#### Internal regulation via heteromerization

Besides the regulation by kinases and phosphatases another member of the *Shaker*-like family alters the functionality of AKT1: AtKC1. AtKC1 is known as regulatory or silent α-subunit of K_in_ *Shaker*-like channels as it shows no currents in *Xenopus* oocytes when expressed alone and affects only K_in_ channels (Dreyer et al., 1997; Jeanguenin et al., 2011). Nevertheless, its participation in K^+^ uptake and its connection to AKT1 has been recognized since long (Reintanz et al., 2002; Pilot et al., 2003). Duby et al. (2008) demonstrated AtKC1′s impact on AKT1. They described that AtKC1 shifts the activation threshold of AKT1 toward more negative values. This in turn would avert K^+^ efflux through AKT1 under unfavorable conditions. The reduction of potential outward currents prevents the plant from K^+^ loss under low K^+^ concentrations. However, the cost of such “a valve” is a reduced channel activity that in turn implies decreased K^+^ influx under more favourable conditions. Geiger et al. (2009) supported, further broadened and fine-tuned this “valve” hypothesis. They showed in electrophysiological experiments the effect of AtKC1 on AKT1 inward and outward currents under varying K^+^ concentrations. Besides affecting the activation threshold, also the K^+^ dependent stability of the pore has been altered in AKT1-AtKC1 heteromers. When the external K^+^ concentration drops, the permeation pathway of K^+^ channels gets instable and collapses (Zhou et al., 2001). The threshold concentration, below which this happens, appears to be a characteristic feature of each channel. Geiger et al. (2009) found that the pore of AKT1-AtKC1 heteromers collapses at higher K^+^ concentrations than that of AKT1 homomers. Thus, heteromers comprise a more efficient block of the K^+^ passage in the unfavorable outward direction.

On top of that, the association of CIPK23 with the heteromeric AKT1-AtKC1 channel has been suggested from interaction analyses in yeast (Grefen and Blatt, 2012) along with an impact of the membrane vesicle trafficking SNARE protein SYP121 (Honsbein et al., 2009). In contrast to CIPK23, SYP121 binds only to the AtKC1 α-subunit but not to the AKT1 α-subunit. SNARE proteins are involved in vesicle targeting and fusion. Thus, K^+^ transport is not only regulated via the channel activity but also by membrane trafficking processes. Interestingly, the transcript level of AKT1 is constant under different environmental conditions (Lagarde et al., 1996; Pilot et al., 2003). But, the expression levels of its regulators change according to environmental stimuli (Pilot et al., 2003, review: Batistic and Kudla, 2004; Tripathi et al., 2009).

### Outward rectifiers in roots

#### GORK in root hairs

Alongside the inward rectifying K^+^ channel AKT1, the outward rectifying K^+^ channel GORK is expressed in root epidermal cells (Ivashikina et al., 2001). GORK activates upon membrane depolarization and its gating depends on the extracellular K^+^ concentration. Environmental changes in the surrounding of root hairs can appear rapidly and in response the membrane depolarizes (Càrdenas et al., 2000). GORK activates under these conditions and is considered to initiate the repolarization of the membrane. By controlling the membrane potential and the turgor in root hairs, the plant can react on environmental changes, like absence or abundance of water that cause changes in solute concentrations and affect the mechanical stability and the hydration status of the root. Furthermore, the ability of GORK to sense the extracellular K^+^ concentration is supposed to enable the root hair to sense and flexibly react on the K^+^ content in the soil.

#### Root to shoot communication via SKOR

K^+^ is transported from roots to the upper parts of the plant via the xylem. The outward rectifying *Shaker*-like channel SKOR is expressed in the pericycle and the xylem parenchyma in roots. SKOR was identified as transport protein responsible for loading K^+^ to the xylem based on the finding that its disruption strongly reduced the K^+^ content in the shoot while the K^+^ content in roots remained unaffected (Gaymard et al., 1998).

In addition to the membrane voltage, SKOR is modulated by the external K^+^ concentration. In the presence of ample external K^+^, the channel needs a higher membrane voltage to open and thus minimizes the risk to serve as an undesirable K^+^-influx pathway. Such behavior is achieved by a complex interplay between the pore region and the last transmembrane domain of the channel that is responsible for final channel opening and closure. When the external K^+^ concentration is high, the pore region is quite rigid and strongly interacts with the last transmembrane domain of the channel. As a consequence the channel is stabilized in a closed state. Under low external K^+^ conditions the pore region is less occupied by K^+^ ions. As a consequence, the pore is more flexible and does not interact with the surrounding transmembrane domains anymore. Opening of the channel is possible with less energy input, i.e., at less positive membrane voltages. If the last transmembrane domains rearrange and unclench the conduction pathway, intracellular K^+^ ions can re-enter the pore, stabilize it in a permeable conformation and thus enable a K^+^ outward current (the K^+^-sensing mechanisms has been animated in the supplementary material of Johansson et al., 2006).

K^+^ distribution is also influenced by factors that are involved in stress signaling. SKOR expression is inhibited by abscisic acid (ABA). It was proposed that the reduced K^+^ release to the xylem in response to ABA could be a possibility to adjust osmotic conditions by roots in stress situations (Gaymard et al., 1998). Besides, intra- and extracellular acidification negatively affects the SKOR currents. As the regulation via ABA appears on the transcriptional level, the pH sensitivity might be a complementary process to prevent K^+^ loss from roots toward the shoot tissue (Lacombe et al., 2000a).

Hydrogen peroxide (H_2_O_2_) exhibits a contrary effect on SKOR currents. Reactive oxygen species function as signal and regulator in plant development and in responses to environmental stress situations (Torres and Dangl, 2005; Gapper and Dolan, 2006). Treatment with H_2_O_2_ leads to an increase in SKOR outward currents and a decrease in its half activation time (Garcia-Mata et al., 2010). This finding points to a relation between reactive oxygen species and K^+^ partitioning during developmental processes and stress responses.

### Phloem-allocation and retrieval

Once loaded into the xylem, K^+^ circulates within the whole plant. There, other K^+^ channels contribute to the further distribution. The *Shaker*-like potassium channel AKT2^2^ is mainly expressed in the vascular tissue of aerial parts and in guard cells of plants. However, it is not expressed until the plant is widely independent of carbohydrates provided by the seed (Marten et al., 1999; Deeken et al., 2000; Lacombe et al., 2000b; Szyroki et al., 2001; Ivashikina et al., 2005).

#### Charging and using the potassium battery

As the only member of the *Shaker*-like channels in plants, AKT2 features a unique channel property and can mediate both, inward and outward K^+^ currents. AKT2 is in fact a specialized inward rectifying channel that can be changed into a non-rectifying channel. It exhibits two phosphorylation status-dependent gating modes that are inter-convertible (Dreyer et al., 2001; Chèrel et al., 2002; Michard et al., 2005a,b). The non-phosphorylated AKT2 (mode 1) is lacking its outward component and behaves like an inward rectifying channel. In contrast, the phosphorylated AKT2 (mode 2) is permanently open and able to conduct K^+^ in the inward and in the outward direction. Two serine residues located near the intracellular side of the channel are identified as targets for phosphorylation (Michard et al., 2005a). Nevertheless, it is proposed that the two phospho-serine residues alone are not sufficient to completely convert AKT2 between its modes. Sandmann et al. (2011) proposed rather a transition via a cascade of posttranslational (so far unknown) modifications. This hypothesis is fuelled by experimental observations. A lysine within the voltage sensor enables AKT2 to sense its phosphorylation status and to change between the two modes. Replacement of the lysine by serine or arginine keeps AKT2 in its inward rectifying mode 1 (Michard et al., 2005b; Sandmann et al., 2011).

Summing up, AKT2 can modulate the membrane voltage by switching between its modes of an inward or a non-rectifying channel, respectively, and phosphorylation acts as a tool for fine tuning (Deeken et al., 2002; Michard et al., 2005a,b). Gajdanowicz et al. (2011) embedded AKT2 as a central player in a “potassium battery” model in which K^+^ serves as mobile energy source in vascular tissues. In source tissues, the plant invests energy to load K^+^ into the phloem sieve element companion cell complexes. The loaded potassium is then circulating with the phloem stream. Under energy limiting conditions, the AKT2 channel can be switched from its inward-rectifying to its non-rectifying mode and thus enables a passage for K^+^ efflux. This in turn enables the use of the K^+^ gradient between the phloem and the apoplast for the reloading of photoassimilates into the phloem. This “potassium battery” concept is illustrated in the supplementary material of Gajdanowicz et al. (2011). Limiting conditions occur for example under ATP shortage or when the H^+^-ATPase is down-regulated by cellular signals. The normally used H^+^ gradient is then complemented by the K^+^ gradient. Besides tapping the “battery,” the AKT2 channel is also proposed to charge it depending on its actual gating mode (Michard et al., 2005b).

#### Further effects on AKT2

In addition to the gating mode modulations, AKT2 was also demonstrated to act on diverse signals involved in stress responses. The expression level of AKT2 increases in the presence of ABA, light and CO_2_ assimilates (Deeken et al., 2000; Lacombe et al., 2000b). Primarily, the influences of the last two factors led to the view that AKT2 plays a role in phloem transport.

Macroscopic K^+^currents mediated by AKT2 are modulated by changes in internal and external pH and external Ca^2+^ (Marten et al., 1999; Lacombe et al., 2000b). While external Ca^2+^ blocks inward currents at negative voltages in a voltage-dependent manner, acidification on both sides of the membrane diminishes AKT2 currents in the whole voltage range. Changes in pH and Ca^2+^ do not affect the gating mode of the channel indicating that H^+^ and Ca^2+^ affect only the permeation pathway of AKT2. The sensitivity of AKT2 toward Ca^2+^ was investigated in guard cells. Ivashikina et al. (2005) showed in experiments on guard cell protoplasts that the Ca^2+^ sensitivity of K^+^ uptake channels correlates with the presence of AKT2 subunits.

Recently, Held et al. (2011) demonstrated the association of AKT2 with CIPK6 and CBL4 and the effect of this assembly on macroscopic AKT2 currents. In contrast to the AKT1-CIPK-CBL complexes, no phosphorylation events could be detected *in vitro* so far. Held et al. therefore proposed for these findings a Ca^2+^ dependent targeting of AKT2 to the plasma membrane that depends solely on the physical interaction of AKT2 with CIPK6/CBL4 rather than a regulation of the channel via phosphorylation.

### Guard cells and its K^+^ channel population

**Figure 3:**
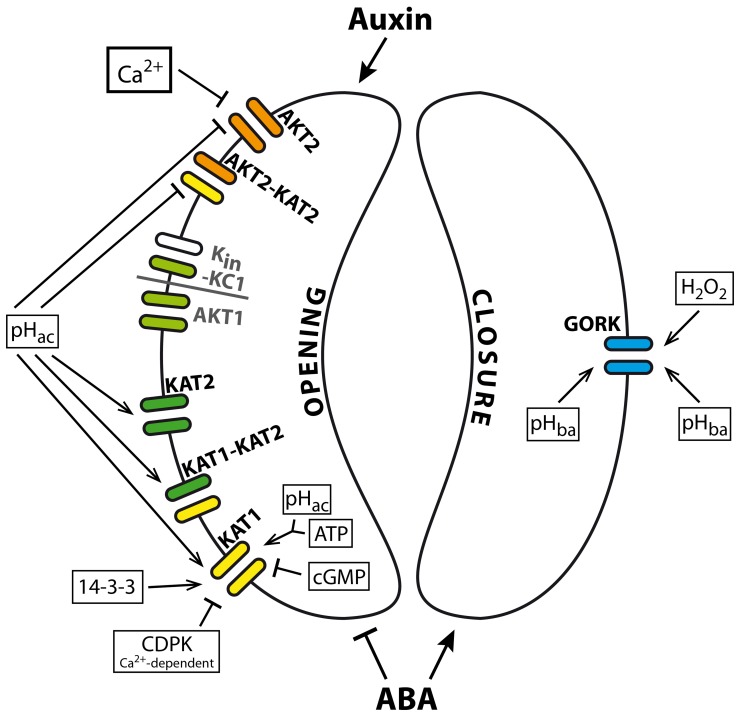
**K^+^ channels in *Arabidopsis* guard cells and their effectors**. Changes in membrane potential lead to stomatal opening or closure, respectively. Membrane hyperpolarisation in response to H^+^-ATPase activity activates inward-rectifying K^+^ channels. Transcripts for KAT1, KAT2, AKT1, AKT2, and AtKC1 are detectable in guard cells (Szyroki et al., 2001). All these K^+^ channel subunits form heterotetrameric channels like KAT1-KAT2, AKT2-KAT2, and AKT1-AtKC1. The impact of AKT1 and K_in_-AtKC1 on stomatal movement has not been investigated in detail. Acidification affects directly the currents through K^+^ channels in guard cells. K_in_ channels are activated upon extracellular acidification, while currents through K_weak_ channels decrease. Besides, the K_weak_ channel AKT2 is negatively affected by extracellular Ca^2+^. KAT1 currents are furthermore modulated by intracellular pH, ATP, and cGMP. ATP and cGMP have antagonistic effects. Moreover, stomatal K^+^ channels are affected by signals via signal transduction cascades. Effects of 14-3-3 proteins, Ca^2+^ and kinases have been reported. Membrane depolarization, on the other hand, caused by the inactivation of H^+^-ATPases and activation of anion channels activates the K_out_ channel GORK. GORK currents are positively influenced by extra- and intra-cellular alkalinisation. Furthermore, the current enhancing effect of H_2_O_2_ is under investigation. Both, stomatal opening and closure are affected by phytohormones. While Auxin evokes stomatal opening, ABA inhibits its opening but evokes closure of stomata. Abbreviations: pH_ac_, acidification; pH_ba_, alkalinisation.

Two third of the *Shaker* channel family members are expressed in guard cells. Besides AKT2, also KAT1, KAT2, AKT1, AtKC1, and GORK are detectable there and have important impacts on stomatal opening and closure (Szyroki et al., 2001; Ivashikina et al., 2005; Lebaudy et al., 2008b). Vast signal transduction pathways coordinate stomatal movement. In case of stomatal opening, they result in the activation of K_in_ channels and the uptake of K^+^and anions, which finally leads to an increase of guard cell turgor. In case of stomatal closure, K_out_ channels are activated, K^+^ is released together with anions, water is passively flowing out and guard cell turgor decreases. Recent comprehensive reviews of signal transduction pathways that affect stomatal opening and closure were published by Pandey et al. (2007) or Kim et al. (2010). Figure 3 shows an overview of the regulation of K^+^ channels involved in stomatal movements.

#### Channel variability in guard cells

Although KAT1 represents the dominant K_in_ channel in guard cells, it is not essential for stomatal opening (Ichida et al., 1997; Kwak et al., 2001; Szyroki et al., 2001). The coevally expressed K_in_ channel subunits AKT1, AKT2, and KAT2 are able to compensate for the loss of KAT1. The expression pattern of all guard cell K_in_ channel subunits is not exactly identical as exemplified by KAT1 and KAT2. Both subunits are expressed in guard cells. But, KAT1 is only expressed in guard cells of leaves and petioles, while KAT2 is additionally expressed in guard cells of the stem (Nakamura et al., 1995; Pilot et al., 2001). That points to different available sets of K^+^ channels dependent on the guard cell location. Furthermore, K^+^ channel subunits are able to form heteromeric channels in plants (Dreyer et al., 1997; Lebaudy et al., 2008a). For KAT1-KAT2 heterotetramers it has been shown that their basic properties are similar to properties observed for the homotetrameric KAT1 and KAT2 channels (Pilot et al., 2001; Lebaudy et al., 2010). In contrast, the AKT2-KAT2 heterotetramer combines different properties of its parental channels and forms a new functional type of a K^+^ channel (Xicluna et al., 2007). The gating properties of the heterotetramer are inherited from AKT2, a weak-rectifying K^+^ channel described above. The sensitivities to Ca^2+^ and H^+^ are inherited from KAT2. Thus, K^+^ channel heteromers notably contribute to an increase in channel variability and enhance the regulatory possibilities of K^+^ channels.

#### K_in_ channels contribute to stomatal opening

For activation of K_in_ channels the membrane potential needs to be hyperpolarized. Hyperpolarization is achieved through the activity of H^+^-ATPases that transport protons under ATP consumption out of the cell. The membrane voltage is sensed by the intrinsic voltage sensor that is formed by the transmembrane regions S1–S4. An important role is played especially by the positive charges in S4 (Figure 1A). The four voltage sensors of the channel induce conformational changes in the protein that then result in an opening of the permeation pathway. This voltage-sensitivity is modulated by other factors that interact with the channel protein. Indeed, many experiments show the sensitivity of guard cell K_in_ channels to changes in pH (Hedrich et al., 1995; Hoshi, 1995; Marten et al., 1999; Pilot et al., 2001; Xicluna et al., 2007). KAT1, KAT2 and the heteromeric KAT1-KAT2 are activated by extracellular and intracellular acidification due to a shift of the voltage dependence of the channels to more positive values. A histidine residue conserved among plant K_in_ channels that is located in the pore was suggested to sense pH changes in K_in_ channels (Hoth et al., 1997; Hoth and Hedrich, 1999). For KST1, a K_in_ channel from potato guard cells, it has been shown that this histidine is part of the pH sensor. Surprisingly, mutations of this histidine in KAT1 did not affect its pH dependence. Further investigation revealed that KAT1 senses pH changes via a sensory cloud rather than a single residue (Gonzalez et al., 2012). Besides, KAT1 is also modulated by ATP and cGMP. While cGMP reduces KAT1 currents, ATP affects KAT1 positively. Thus, ATP and cGMP show antagonistic effects (Hoshi, 1995).

Another regulator of guard cell K_in_ currents might be extracellular Ca^2+^. Here, AKT2 is the only channel affected directly by external Ca^2+^ (Marten et al., 1999; Latz et al., 2007b). While AKT2 is blocked by Ca^2+^, KAT1, KAT2 and AKT1 do not show any response (Szyroki et al., 2001; Ivashikina et al., 2005; Brüggemann et al., 1999). It is therefore proposed that experimentally observed sensitivity of guard cell K_in_ channels to extracellular Ca^2+^ is conferred by AKT2 subunits (Ivashikina et al., 2005).

Furthermore, effects of regulatory proteins on KAT1 have been shown. For instance, KAT1 is phosphorylated in a Ca^2+^-dependent manner in the presence of CDPK-a Ca^2+^-dependent protein kinase with a calmodulin-like domain (Li et al., 1998). This study used a recombinant CDPK from the bean *Vicia faba* and did not show whether KAT1 is phosphorylated directly by CDPK or rather other proteins are affected by the kinase. Berkowitz et al. (2000) showed in electrophysiological experiments that the recombinant CDPK has a negative effect on KAT1 currents. Ca^2+^-dependent phosphorylation of KAT1 is further supported by a study that manipulated a protein kinase C (PKC) present in *X. laevis* oocytes (Sato et al., 2010). Upon activation of PKC that has similar target sites as plant Ca^2+^-dependent kinases, KAT1 currents decline. In addition, recombinant 14-3-3 proteins from maize stimulated KAT1 currents (Sottocornola et al., 2006, 2008). These studies provide a first glimpse on the broad range of feasible effectors of K^+^ channels in guard cells.

Additionally it was found that the channel population within the membrane undergoes regulation as well (Mikosch et al., 2006, 2009; Sutter et al., 2006, 2007; Sieben et al., 2008; Reuff et al., 2010). It has been shown that KAT1 interacts with SNARE proteins (see above), and ABA triggers endocytosis of KAT1 from the plasma membrane. Furthermore, the ER export motif of KAT1 subunits is important for proper channel trafficking. It has been shown that efficient ER export of KAT1 depends on an acidic motif in the C-terminus. Therefore, endo- and exo-cytosis, as well as the ER export of K^+^ channels might be another level for regulating channel densities and K^+^ currents across the membrane.

#### K_out_ channels during stomatal closure

K_out_ channels are activated upon depolarization. Such a membrane voltage change is achieved by inhibition of the H^+^-ATPase and activation of anion channels. GORK is the only K_out_ channel identified in guard cells and is responsible for stomatal closure (Szyroki et al., 2001; Hosy et al., 2003). In contrast to inward rectifying channels, GORK currents are reduced with decreasing internal and external pH (Blatt, 1992; Ache et al., 2000). GORK also senses the external K^+^ concentration, so that at higher external K^+^ it requires more positive voltage for its activation (Ache et al., 2000). A similar analogy to SKOR might also hold for the direct interaction of GORK with the stress signaling molecule H_2_O_2_. GORK and SKOR share the cysteine residue that has been shown to be responsible for the activation effect in SKOR (Garcia-Mata et al., 2010). Nevertheless, the impact of H_2_O_2_ on GORK and the role of this presumed regulation in guard cell physiology still need to be investigated. Earlier reports have shown that H_2_O_2_ is an important player in stomatal signaling (reviewed by Wang and Song, 2008).

Alongside the activation of K_out_ channels during stomatal closure, K_in_ channels are deactivated (Blatt, 1990; Thiel et al., 1992). A knock-out mutant of the K_in_ channel AKT1 has been shown to be more resistant toward water stress than wild type plants (Nieves-Cordones et al., 2012). Transpiration was reduced and stomata closure was more efficient in knock-out plants treated with ABA. Thus, the inactivation of K_in_ channels favors stomatal closure but it is not essential for the process of closure itself (MacRobbie, 1998). Furthermore, very similar phenotypes of *akt1* and *cipk23* knock-out plants could be observed suggesting a regulation of AKT1 by CIPK23 also in guard cells as it has been shown already for AKT1 in roots.

#### Influences of phytohormones

The phytohormones auxin and ABA cause opposing effects on stomata. Auxin is involved in plant developmental processes and promotes stomatal opening, whereas, ABA is involved in various stress responses. It prevents the opening and promotes the closure of stomata (Gehring et al., 1990). The direct influence of ABA on guard cell K^+^ currents was shown by Blatt and Armstrong (1993). ABA treatment leads to inactivation of K_in_ and activation of K_out_ channels in guard cells. Although the phytohormon affects the transcription level of the K_out_ channel GORK in roots and shoots, the transcript level in guard cells remains unaffected (Becker et al., 2003). Besides, electrophysiological analyses exclude the direct effect of ABA on outward currents in guard cells. Therefore, ABA seems to affect guard cell K_out_ currents indirectly. As ABA signals from roots come along with alkalinisation of the guard cell cytoplasm (Blatt and Armstrong, 1993), the pH sensitive GORK can be activated and affected by ABA by this long distance signaling pathway (Blatt, 1992; Ache et al., 2000; Becker et al., 2003).

Auxin, on the other hand, stimulates the transcription of *KAT1* and *KAT2* (Philippar et al., 2004). It is not clear, however, whether this stimulation is tissue specific as in the case of GORK or whether it is a general feature of these K_in_ channel genes in different parts of the plant.

### Influence on pollen tube development

The *Shaker* channel SPIK is the main K_in_ channel in pollen and exclusively expressed there (Mouline et al., 2002; Zhao et al., 2013). Its disruption affects negatively pollen tube growth. The activity of SPIK is enhanced by decreasing external pH and negatively affected by the Ca^2+^-dependent protein kinases CDPK11 and CDPK24. Ca^2+^ affects K_in_ currents only in the pollen tube but not in pollen grain protoplasts. It has been shown that the effect of Ca^2+^ is dependent on the presence of both kinases. In the absence of one of the kinases Ca^2+^ cannot block pollen K_in_ currents. Zhao and colleagues propose that Ca^2+^ acts negatively on SPIK via a kinase cascade, in which CDPK11 phosphorylates CDPK24.

## Conclusions

K^+^ channels are important for K^+^ uptake from the soil, its distribution within the plant and processes to maintain and support plant growth. The past two decades revealed crucial information especially for plant *Shaker* like channels regarding the structure, physiological role and-to a minor extent-regarding their regulation. In contrast, our knowledge on TPK channels is far more rudimentary. The challenge of the future of plant K^+^ channel research will be to identify the complex regulatory networks that regulate their activity and to understand the dynamics of these networks.

### Conflict of interest statement

The authors declare that the research was conducted in the absence of any commercial or financial relationships that could be construed as a potential conflict of interest.

## Footnotes

^1^pS/pT indicate the potential phosphorylation of the serine or threonine residue, respectively.

^2^In literature, the gene encoded by the locus At4g22200 has been named AKT2, AKT3 and AKT2/3. To avoid confusions, we will summarize the data under the name AKT2 irrespective of the alternative names used in the original publications.

## Abbreviations

ABA: abscisic acid
ATP: adenosine triphosphate
CBL: calcineurin B-like calcium sensor
CDPK: Ca^2+^ dependent protein kinase
cGMP: cyclic guanosine monophosphate
CIPK: CBL-interacting protein kinase
ER: endoplasmic reticulum
GFP: green fluorescent protein
P: pore
PKC: protein kinase C
PP2C: 2C-type protein phosphatase
SNARE: soluble N-ethylmaleimide–sensitive factor protein attachment protein receptor
TM: transmembrane
YFP: yellow fluorescent protein.
